# The role of cullin4B in human cancers

**DOI:** 10.1186/s40164-017-0077-2

**Published:** 2017-06-15

**Authors:** Ying Li, Xin Wang

**Affiliations:** 1 Department of Hematology, Shandong Provincial Hospital Affiliated to Shandong University, No.324, Jingwu Road, Jinan, 250021 Shandong People’s Republic of China; 2 Shandong University School of Medicine, Jinan, 250012 Shandong People’s Republic of China

**Keywords:** Cullin4B, Cancer, Cell cycle, Post-transcriptional modification

## Abstract

Cullin 4B (CUL4B) is a scaffold of the Cullin4B-Ring E3 ligase complex (CRL4B) that plays an important role in proteolysis and is implicated in tumorigenesis. Aberrant expression of CUL4B has been reported in various types of human diseases. Recently, studies have shown that CUL4B was overexpressed in a multitude of solid neoplasms and affect the expression of several tumor suppressor genes. In this review, we aim to summarize the biological function of CUL4B in order to better understand its pathogenesis in human cancers.

## Background

Cullins are evolutionarily highly conserved protein molecule family in most mammals. There are eight subtypes cullins in human genome, including cullin1, cullin2, cullin3, cullin4A, cullin4B, cullin5, cullin7 and PARC, also known cullin9 [1]. The cullin protein functions as a scaffold in the largest class of really interesting new gene (RING) E3 ligases, by binding to RING box proteins through a highly conserved homology domain. Cullins play a key role in selective degradation of various proteins which are involved in versatile cell biology behaviors, including cell cycle and signaling regulation. More recently, studies have focused on their connections with human diseases, including cancer [2–4].

There are two members in cullin4 (CUL4) family, CUL4A and CUL4B. CUL4B is highly sequence homology with CUL4A, sharing 83% sequence identity [5]. Scientists used to think that CUL4A and CUL4B involved several cell life activities in an almost similar way. Nevertheless, latest publications indicate that there are big differences in functions and specific mechanisms between CUL4A and CUL4B. Difference from CUL4A and other cullins that it carries their nuclear localization signal (NLS) in their C termini, whereas NLS in CUL4B is located in its N terminus [5]. CUL4A and CUL4B can interact with DDB21, a substrate adaptor, and they may target the same substrates and function redundantly in some cellular functions, such as genome integrity maintenance, DNA replication and cell cycle regulation [6–8]. However, CUL4B has been demonstrated to target substrates, such as WD repeat containing protein5 (WDR5) and peroxiredoxin III (PrxIII), that are not targeted by CUL4A [9–11]. CUL4B knockout mice were embryonic lethal [12, 13]. In contrast, except for failure in spermatogenesis, CUL4B knockout mice have no remarkable abnormalities [14, 15]. CUL4A is highly expressed in the pancreas, testes and in T cells [16], while CUL4B is most highly expressed in pancreatic tissue, endocrine glands, the cerebellum, the lower GI tract, bone marrow and the testes [17].

**Fig. 1:**
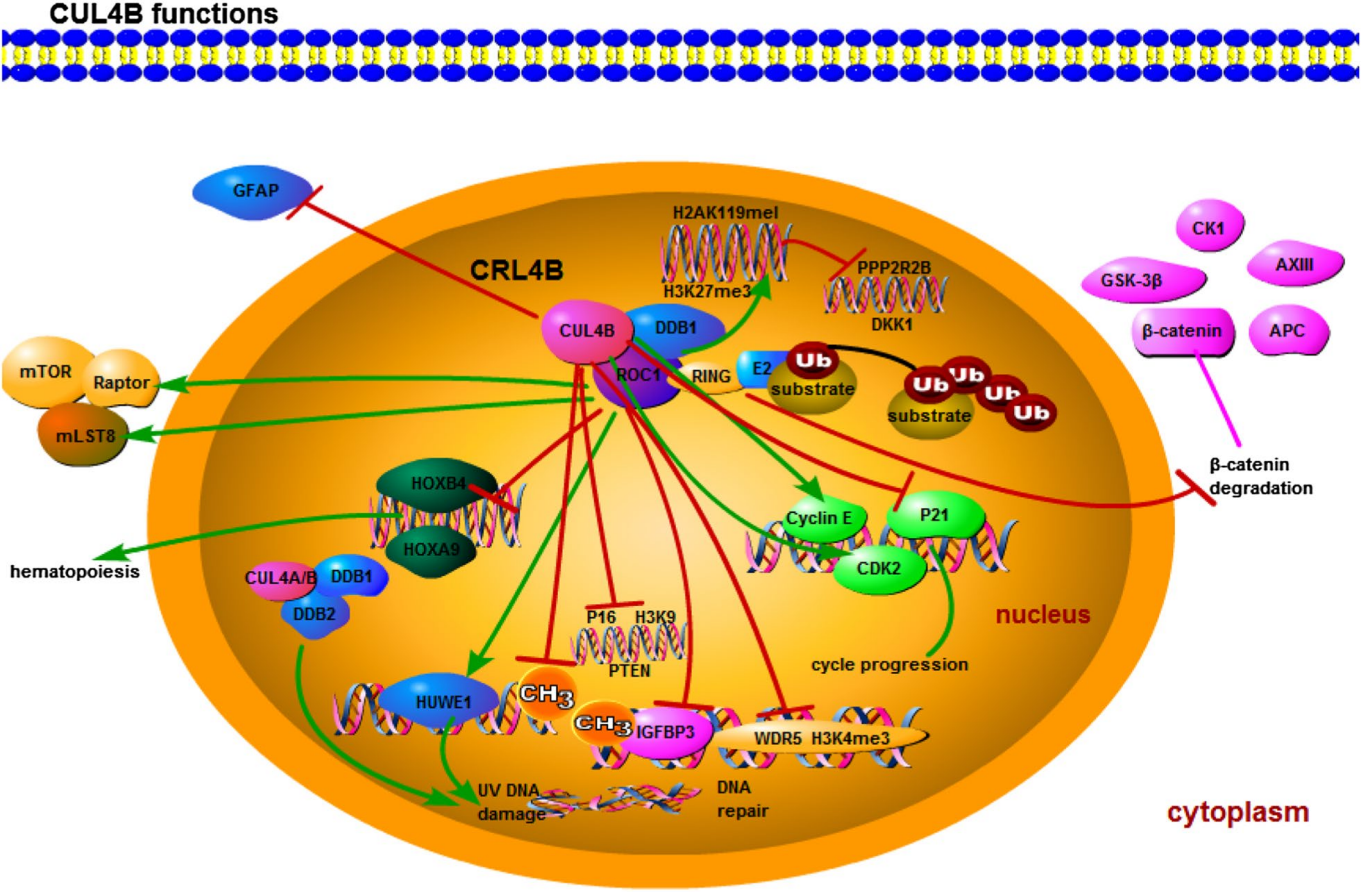
The schematic representation of CUL4B and associated factors

Recently, the function of CUL4B in solid tumors has been gradually uncovered and attracts a lot of interests. Plenty of studies have demonstrated that the expression of CUL4B is abnormal in a wide variety of diseases and physiological process (Table 1) [3, 18–21]. Through upregulating or decreasing the expression of CUL4B in cancer cells, these studies have demonstrated clearly that CUL4B acts a pivotal part in cell proliferation, DNA damage and repair, cell cycle progression, metastasis, invasion, DNA methylation and histone acetylation modification, as well as signaling pathways. Furthermore, CUL4B may present potential diagnostic value for human diseases. This review intends to summarize recent insights into CUL4B functions (Fig. 1). In addition, we revealed the findings demonstrating CUL4B’s association with oncogenesis and its important function as bio-marker for tumor diagnosis.

**Table 1:** Studies on CUL4B in diverse diseases and physiological process

Condition or process	First author/s, year (ref.)
X-linked intellectual disability	Zou et al. 2007 [23], Tarpey et al. 2007 [22], Nakagawa et al. 2011 [11], Badura-Stronka [60], Wang et al. 2013 [61], He et al. 2013 [4], Vulto-van et al. 2015 [25]
Spermatogenesis	Yin et al. [62], Kerzendorfer et al. 2011 [63]
Embryogenesis	Cang et al. 2006 [64], Liuet al. 2009 [14], Chen et al. 2012 [7], Jiang et al. 2012 [12]
Cervical carcinoma	Hu et al. 2012 [3], Yang et al. 2015 [20]
Osteosarcoma	Chen et al. 2014 [21]
Colorectal cancer	Hu et al. 2012 [3], Jiang et al. 2013 [27], Song et al. 2015 [54]
Ovarian cancer	Hu et al. 2012 [3], Pan et al. 2013 [65]
Pancreatic cancer	Hu et al. 2012 [3]
Stomach cancer	Hu et al. 2012 [3]
Thyroid cancer	Hu et al. 2012 [3]
Esophageal cancer	Hu et al. 2012 [3]
Kidney cancer	Hu et al. 2012 [3]
Liver cancer	Yuan et al. 2015 [19, 66], Mok et al. 2015 [67], Qu et al. 2016 [68]
Glioma	Dong et al. 2015 [26]
Lung cancer	Hu et al. 2012 [3], Wang et al. 2016 [28], Mi et al. 2017 [18], Jia et al. 2017 [69]

## CUL4B expression in central nervous system

In 2007, Tarpey et al. [22] and Zou et al. [23] noticed that mutations in CUL4B caused an x-linked mental retardation syndrome (XLMR). Individuals with XLMR show obvious clinical features, including growth retardation, mental retardation, relative macrocephaly, truncal obesity and hypogonadism [22, 23]. XLMR-linked CUL4B mutations induced the accumulation of WDR5 and the activation of neuronal genes which could promote neurite extension [24]. Moreover, one of the CUL4B specific substrate, PrxIII, has been reported to affect neural development via regulating the level of reactive oxygen species (ROS) [10]. When CUL4B was silenced, the accumulation of PrxIII inhibited the generation of ROS and enhanced resistance to hypoxia and H2O2-induced apoptosis, and then interfered with cell proliferation and normal cellular functions [10].

Vulto-van Silfhout et al. [25] also discovered that CUL4B was necessary for brain development. They provided first evidence that there was a firm association between CUL4B variants and cerebral malformations. The interaction between CUL4B and WDR62 might contribute to the development of cerebral malformations in patients with CUL4B variants [25]. Furthermore, observation of abnormal neuronal organization in the hippocampus in CUL4B knock-out mice testified that CUL4B played an important role in brain development [7]. In the CUL4B knock-out mice, exons 4-5 of CUL4B in the X chromosome were excised by Sox2-Cre [7]. CUL4B knock-out mice presented hippocampus-related spatial learning and memory deficiencies, while presenting normal appearances [7].

Dong et al. [26] demonstrated that CUL4B might function as an oncogene in malignant glioma tumorigenesis. This paper showed that knockdown CUL4B significantly decreased cell proliferation via inducing G1 phase cell cycle arrest and inhibited the tumor growth in xenografts in nude mice, which revealed that CUL4B knockdown might alleviated glioma tumorigenesis [26].

## CUL4B expression in human cancers

Immunohistochemical staining in esophageal, lung, gastric, colon, pancreatic, cervical, renal, liver, and bladder tumor and matched tissues, showed a significant overexpression of CUL4B in tumor tissue [3, 18–20]. Higher expression of CUL4B in esophageal cancerous tissues has a closely association with lower histological grades [3]. Yang et al. [20] reported that CUL4B was frequently increased in 64 human cervical carcinoma samples compared to 30 adjacent non-tumor cervical tissues. They also found a positive correlation between CUL4B expression and cervical carcinoma histological grades. Meanwhile, CUL4B mRNA level was also higher in 12 of 15 selected paired samples of each grade cancers [20].

High expression of CUL4B expression was reported to be related to tumor histological differentiation, invasion, distant metastasis, tumor size, lymph node metastasis, overall and recurrence rate, as well as disease-free survival in breast, colorectal, cervical, oesophageal and lung cancers [3, 18, 20, 27]. Study has shown that knockdown CUL4B could suppress the epithelial–mesenchymal transition (EMT) progress [28]. However, the mechanism is still unclear. Based on the above facts, CUL4B is generally overexpressed in the cancer tissues and is associated with unfavorable prognosis, which may be a novel tumor marker for tumor diagnosis and prognosis. Further studies are needed to examine the relationship between CUL4B and other non-solid malignancies.

## CUL4B and DNA damage and repair

It has been identified that the CRLs formed by several members of cullin family are involved in DNA damage and repair procedures. Hereinto, CUL4A participated in nucleotide excision repair by CUL4A-DDBl-CSA complex or CUL4A-DDBl-DDB2 complex [29]. Homology of CUL4B and CUL4A is high and only one kind of CUL4 is expressed in primary eukaryotes [5]. Compared with CUL4A, CUL4B possess specific amino acid end consisting of 149 amino acids [30]. Study has certified that CUL4 was engaged in DNA homogenous recombination repair in a way of DDBl-CUL4^CDT2^ complex [31]. Guerrero-Santoro et al. [32] manifested that both DDBl-Cul4A^DDB2^ and DDBl-CUL4B^DDB2^ recognized UV-damaged DNA in the same DDB2-dependent manner. While, the latter is more efficient than the former in monoubiquitinating histone H2A. CUL4B promotes the transfer of DDB1 into the nucleus independently of DDB2 after UV irradiation [32]. CUL4B can regulate the DNA damage induced by UV irradiation.

Further, DNA double strands break increased after treatment of camptothecin (CPT) in lymphoblastic cells conveying CUL4B mutation [31]. Cells from patients with mutant CUL4B were more sensitive to camptothecin (CPT), which indicated that CUL4B may impair CPT-induced topoisomerase I (Topo I) degradation and ubiquitination [31]. Consistent with this, these cells exhibited increased levels of CPT-induced DNA breaks. It has been reported that downregulation of HUWE1 in response to DNA damage is accompanied by the activation of CUL4B and CRL4B was required for proteasomal degradation of HUWE1 [33]. These evidence manifests that CUL4B is associated with DNA damage repair.

## CUL4B and cell cycle progression

Cell cycle is important to maintain tumor or normal cells growth. Lots of studies have shown that deregulation of cell cycle progression could make cells eventually develop as tumor. Cell cycle progression is strictly controlled by multiple mechanisms to ensure its strict and orderly progress. Cyclin-dependent kinases (CDKs), CDK inhibitors (CKIs) and Cyclins are main regulators in cell cycle [34, 35]. Accordingly, maintaining the balance of the above proteins is very necessary.

Cyclin E is important in cell cycle which can bind to CDK2 to regulate G1-S transition and guarantee DNA replication accuracy [36]. Cyclin E alteration has been reported in many cancers and is associated with pathogenesis of malignancies [37–39]. Several papers have shown that cyclin E is regulated by ubiquitin-mediated proteolysis system [40–42]. Knockdown CUL4B induces significant accumulation of cyclin E [5, 42] and a prolonged S phase, resulting in an inhibition of cell proliferation [5]. In conclusion, these studies are suggestive of CUL4B probably targeting cyclin E for degradation to inhibit cell proliferation [5].

P21, one kind of CKIs, can regulate cell cycle by inhibiting the kinase activity of CDK2. Many studies have manifested that p21 accumulates during G1 phase but degraded during S phase [43]. CUL4B negatively regulates the function of p21 by transcriptional repression (repressing the transcription of CDKN1A which encodes p21), but not by proteolysis [44]. CUL4B promotes cell proliferation at least partially through repressing the transcription of Cdkn1a which encodes p21. Silencing of CUL4B expression caused accumulation of p21 protein in extra-embryonic cells, HEK293 and HeLa cells [13, 44].

CUL4B can also upregulate DNA replication by positively regulating CDC6 [8]. Study proves that CDC6 is essential for the formation of pre-replication complexes (pre-RC) [31, 32]. CDK2 is responsible for the stabilization and phosphorylation of CDC6. CULB upregulates the CDK2 expression by negatively transcription in miR-372/373, resulting in the positive regulation of CDC6.

## CUL4B in post-transcriptional modification

Epigenetics is the study of gene post-transcriptional modification. Epigenetic modifications are important for the development and progression of tumors [45]. As research continues, epigenetics has evoked widespread attention to their functions in the regulation of gene expression, including promoter–enhancer interactions, histone modifications, DNA methylation, and noncoding RNA-mediated regulation. The coordinated operation of aspects above is involved in cell cycle regulation, proliferation, apoptosis and so on, and relatives to the ultimate responses in human health and disease [46]. Recently, DNA methylation and histone modifications have been reported in various cancers, and some of which s are found to be associated with poor prognosis.

DNA methylation is one of the most common epigenetic events in eukaryotic cell. It has been revealed that DNA methylation plays a key regulatory role in normal development. Studies have revealed that CUL4B could regulate gene expression by DNA methylation. Study in XLMR shows that eliminated CUL4B expression in nervous cell resulted in the accumulation of WDR5 functioning as a critical substrate of CUL4B and increased the level of H3K4me3 on the neuronal gene promoters [11]. Therefore, CUL4B could degrade via ubiquitination of H3K4 methyltransferase component WDR5 to participate in the transcriptional regulation of epigenetic.

Hu et al. [3] identified CUL4B could play a role of epigenetic transcriptional regulation in gene expression. In order to clarify the mechanistic role of CUL4B, the proteins combined with CUL4B were identified by using affinity purification and mass spectrometry [3]. They demonstrated that CUL4B promoted PRC2 to bind target gene promoters and promoted PRC2 catalyzing the methylation of H3K27. Knockdown CUL4B resulted in loss of H2AK119 monoubiquitination and H3K27 trimethylation, leading to many genes abnormal epigenetic transcription including some suppressor genes such as p16, PTEN [3].

CRL4B plays a crucial role in the recruitment and stabilization of SUV39H1/HP1/DNMT3A onto IGFBP3 promoters [20]. This revealed that the abundance of CpG islands located within the IGFBP3 promoter region were significantly hypomethylated in CUL4B-depleted cells. All of these provide a powerful evidence of that CUL4B is required for the maintenance of epigenetic silencing of target genes [20].

The histone acetyltransferases (HAT) and histone deacetylases (HDAC) are two kinds of enzymes that regulate acetylation and deacetylation [47, 48]. HAT and HDAC are two kinds of important mechanisms in gene transcription regulation. HAT is associated with the activation of gene transcription, while HDAC is associated with the gene transcriptional suppression. The latter contributes to the tumor genesis and progression by inhibiting the transcription of tumor suppressor gene, which has become a popular target in the research of antitumor drug. Now, HDAC inhibitors are effective antineoplastic agents. It is widely used in clinical treatment in various malignancies [49].

CUL4B has been reported to regulate gene expression by transcriptional repression [3, 44]. HDAC1 and HDAC2 are involved in CUL4B-mediated transcriptional repression. Interference HDAC1/2 expression and using of TSA (a kind of HDAC inhibitor) could decrease the transcriptional repressive activity of CUL4B [44]. Meanwhile, up-regulation of CUL4B increased DDB1, HDAC1/2 and SIN3A which were binding to the gene promoter, accompany with catalyzing H2AK119 monoubiquitylation and decrease of the levels of histone acetylation [44]. CRL4B and SIN3A-HDAC complexes interact with each other and co-combine with the CDKN1A and CDKN1C promoters [44]. They caused H2AK119 monoubiquitylation and histone deacetylation and repressed target gene expression at the transcription level, and then regulated cell cycle as described above [44]. DNA methylation and histone ubiquitination/methylation in transcription repression prove that CUL4B may function as an oncogene in tumorigenesis. Although studies have shown that the roles of CUL4B in post-transcriptional modification, there are limited studies about epigenetic related roles so far. Further studies are needed to explore other epigenetic related roles of CUL4B.

## CUL4B and apoptosis

Apoptosis is a complementary of cell proliferation in cell life activity [50, 51]. Many studies have found that tumor cell apoptosis is key to tumor therapy [52, 53]. Down-regulation of CUL4B can inhibit cell proliferation, lead to cell cycle S phase arrest and DNA replication abnormalities, which will promote tumor cell apoptosis. Study has shown that silencing of CUL4B inhibited colorectal cancer cells proliferation by inhibiting Wnt/β-catenin signaling pathway and promoted apoptosis in vitro and in vivo experiments [54]. Chen et al. [21] discovered that interference CUL4B gene expression could effectively inhibit the proliferation of osteosarcoma cells and induce apoptosis. Based on these studies, CUL4B may be a potential target for abnormal regulation associated with malignancy. However, it remains to carry out more studies to explore the relationship between CUL4B and cell apoptosis.

## CUL4B related signaling pathways

As we all know, tumorigenesis has closely connections with abnormal signaling pathways. Recently, many studies reported that aberrant CUL4B expression is related to the activation of several signal transduction pathways. Wnt/β-catenin signaling pathway has been demonstrated abnormal activated in many kinds of cancer [55–57]. At present, Yuan et al. [19] found CUL4B functioned as a positively regulator in Wnt/β-catenin signaling in HCC. CUL4B and β-catenin are over-expressed in HCC tissues and have positive correlation. They manifested that β-catenin was downregulated when they knockdown CUL4B in HCC cell lines. Further, they discovered that CUL4B could protect β-catenin from GSK3-mediated degradation and prevent β-catenin degradation by repressing Wnt antagonists. In addition, CRL4B/PRC2 complexes was proved to activate Wnt signaling pathway through promoting H2AK119me1 and H3K27me3 to repress expression of Wnt inhibitors [19].

The PI3K/Akt/mTOR pathway is one of the most recurrently changed signaling pathway in cancers. Its abnormal activation plays a central role in promoting cancer genesis. PTEN, an inhibitor of PI3K signal pathway, is frequently lost in a multitude of cancers. Absent expression of PTEN leads to accumulation of PIP3 and then increases activity of AKT. As known, mTOR is abnormally activated because of PTEN loss, dysregulation of mTOR regulators and aberrant upstream signaling such as PI3K/AKT activation.

CRL4 might influenced mTOR activity potentially by interacting with DDB1, mTOR complex components mLST8 and Raptor [58]. Silence of CUL4B greatly decrease mTOR-mediated S6K1 phosphorylation [58]. Lately, Hu et al. proved that CUL4B knockdown in several different cancer cell lines led to increased expression of p16 and PTEN at the transcriptional level. Taken together, CUL4B may upregulate PI3K/Akt/mTOR pathway in oncogenesis to promote cellular survival, cell cycle progression and growth. However, details still need more investigations.

In order to discover and enhance cancer immunotherapy, abundant studies have focused on the novel examination about harnessing the treatment of cell signaling pathways mediating the tumor-associated immune suppression. Cancer occurrence and progression require a favorable microenvironment. AKT/β-catenin signaling cascades in malignancies are often regarded as therapeutic targets. Recently, Qian et al. [59] demonstrated that accumulation of myeloid-derived suppressor cells (MDSCs) whose presence acts as an important characteristic of tumor-permissive microenvironment, was mediated by downregulation of AKT/β-catenin pathway in CUL4B knock-out mice. CUL4B has previously been reported to be up-expressed in many malignancies and appears to be positively correlated with tumor progression [3, 19–21, 27]. on the contrary, Qian et al. [59] proved that CUL4B acted as a suppressor of tumorigenesis via limiting the partial functions of MDSCs to maintaining the immunosuppressive tumor microenvironment.

## CUL4B and hematopoiesis

Hematopoietic stem cells (HSCs) have the ability to self-renew and produce identical, multipotent daughter HSCs or undergo differentiation to form any and every cell type in the blood system. Hematopoiesis is a tightly regulated, context-dependent process that relies on several intrinsic and extrinsic factors. Many HOX genes, highly conserved regulators, were expressed during the early stages of hematopoiesis within progenitor cells and underwent downregulation as cells differentiate. Paradoxically, some studies announced that constitutive CUL4A- and CUL4B-knockout mice exhibited no defects in hematopoiesis [13]. However, another showed that the deletion of either of the CUL4A nor CUL4B led to increased accumulation of HOXB4, indicating a redundant role for CUL4A and CUL4B in hematopoiesis [16]. It has been indicated that CRL4s played a significant role in regulating the self-renewal and differentiation of hematopoietic stem cells by targeting HOXA9 and HOXB4 for ubiquitination and degradation [16]. Future work is needed to identify the exact and different roles of CUL4A and CUL4B in hematopoiesis.

## Conclusions and future perspectives

In conclusion, aberrant expression of CUL4B was found in several malignant diseases. CUL4B works as an oncogene in most of cancers. CUL4B influents human diseases development and progression by regulating a wide range of cellular processes, such as cell cycle, promoter methylation and histone deacetylation, DNA damage and repair, etc. Downregulation of CUL4B results in inhibited cancer cell proliferation, and it may act as a potential index of diagnose and therapeutic strategy in cancers. On the contrary, CUL4B acts as a tumor suppressor gene in the immunosuppressive tumor microenvironment. Upregulation of CUL4B is closely associated with the activation of many cancer-associated signaling pathways. Since CUL4B can influence human diseases development and progression by regulating a wide range of cellular processes, CUL4B will provide new insights into cancer diagnosis and treatment. However, further investigations are needed to clarify concrete mechanisms of CUL4B in cancers. Consequently, works on CUL4B in human diseases, especially cancers, deserve more concerns in the future.

## Abbreviations

CUL4B: cullin 4B
RING: really interesting new gene
CRL: Cullin -Ring E3 ligase complex
NLS: nuclear localization signal
XLMR: x-linked mental retardation syndrome
CDKs: cyclin-dependent kinases
CKIs: CDK inhibitors
WDR5: WD repeat containing protein 5
H3K4me3: trimethylated H3K4
HAT: histone acetyltransferases
HDAC: histone deacetylases
HCC: hepatocellular carcinoma
MDSCs: myeloid-derived suppressor cells
PrxIII: peroxiredoxin III
ROS: reactive oxygen species
HSCs: hematopoietic stem cells
